# Sexual preference for prepubescent children is associated with enhanced processing of child faces in juveniles

**DOI:** 10.1007/s00787-020-01684-4

**Published:** 2020-11-24

**Authors:** Lara Speer, Miriam Schuler, Julian Keil, James K. Moran, Pierre Pantazidis, Till Amelung, Jakob Florack, Klaus M. Beier, Daniel Senkowski

**Affiliations:** 1 Charité – Universitätsmedizin Berlin, corporate member of Freie Universität Berlin, Humboldt-Universität zu Berlin, and Berlin Institute of Health, Institute of Sexology and Sexual Medicine, Charitéplatz 1, 10117 Berlin, Germany; 2 Department of Psychiatry and Psychotherapy, St. Hedwig Hospital, Charité – Universitätsmedizin Berlin, corporate member of Freie Universität Berlin, Humboldt-Universität zu Berlin, and Berlin Institute of Health, Berlin, Germany; 3 Biological Psychology, Christian-Albrechts-University Kiel, Kiel, Germany; 4 Child and Adolescent Psychiatry, Vivantes Hospital Berlin, Berlin, Germany

**Keywords:** Child sexual abuse, Sexual behavior, Neuroimaging, Pedophilia, Electroencephalography, Event-related potentials

## Abstract

Child sexual abuse offences (CSOs) represent a severe ethical and socioeconomic burden for society. Juveniles with a sexual preference for prepubescent children (PP) commit a large percentage of CSOs, but have been widely neglected in neuroscience research. Aberrant neural responses to face stimuli have been observed in men with pedophilic interest. Thus far, it is unknown whether such aberrations exist already in PP. A passive face-viewing paradigm, including the presentation of child and adult faces, was deployed and high-density electroencephalography data were recorded. The study group comprised 25 PP and the control group involved 22 juveniles with age-adequate sexual preference. Attractiveness ratings and evoked brain responses were obtained for the face stimuli. An aberrant pattern of attractiveness ratings for child vs. adult faces was found in the PP group. Moreover, elevated occipital P1 amplitudes were observed for adult vs. child faces in both groups. At longer latency (340–426 ms), a stronger negative deflection to child vs. adult faces, which was source localized in higher visual, parietal and frontal regions, was specifically observed in the PP group. Our study provides evidence for enhanced neural processing of child face stimuli in PP, which might reflect elevated attention capture of face stimuli depicting members from the sexually preferred age group. This study expands our understanding of the neural foundations underlying sexual interest in prepubescent children and provides a promising path for the uncovering of objective biomarkers of sexual responsiveness to childlike body schemes in juveniles.

## Introduction

In addition to physical injuries, child sexual abuse offences (CSOs) often lead to the development of mental disorders [1] and are associated with an increased likelihood of subsequent criminal behavior, e.g. becoming a child sexual offender [2, 3]. Hence, CSOs represent a serious ethical and socioeconomic burden for society. CSOs are not only committed by adults but also by children and juveniles. For instance, in Germany, children and juveniles constituted about 30% of all reported CSOs [4]. A major risk factor for committing CSOs is the sexual preference for prepubescent children [5]. About 20–50% of adult child sexual offenders report to have a pedophilic sexual preference [6–8], which often manifests during adolescence and is presumably a relatively stable personality trait [9–11]. Therefore, juveniles and adults with sexual preference for childlike body schemes are comparably exposed to a higher risk of committing CSOs. While the socioeconomic and demographic factors contributing to pedophilia have been extensively studied in adults [10, 12, 13], the neural signatures of sexual preference for prepubescent children are not well understood, especially not in juveniles. There are some studies revealing aberrant brain responses of erotic child and/or adult stimuli in adults with pedophilic interest [14–17]. However, only one study in adults has shown aberrant neural processing of face stimuli depicting prepubescent children and adults in men with sexual interest in children [18]. To date, however, it is unknown whether aberrations in the processing of child faces are also found in juveniles with sexual preference for prepubescent children. Addressing this question could help uncover objective biomarkers underlying  the sexual age preference at early development stages.

In clinical practice, sexual preference for prepubescent children (in the sense of pedophilia) is frequently divided into an exclusive type with sexual preference only for prepubescent bodies and a non-exclusive type with sexual responsiveness to the childlike and adult body scheme. Age sexual preference is usually assessed through sexual case history or questionnaires regarding sexual history; masturbatory sexual fantasies; reports by relatives; behavioral observations; measurements of physical responses to sexual stimuli, like eye movements, viewing time or differences in penile volume (for an overview, see Jordan and colleagues [19]). However, these approaches are prone to subjective biases and they can easily be manipulated [5, 20, 21]. For these reasons, a few attempts have been made to quantify the assessment of sexual preference for prepubescent children using neurophysiological measures. For instance, an electroencephalography (EEG) study has examined men with pedophilia and control participants with sexual interest in adults watching erotic stimuli depicting adults [22]. Investigating event-related potentials (ERPs), the authors found reduced frontal P2 amplitudes, indicating reduced responsiveness to the sexually arousing adult stimuli in adult men with pedophilic interest. Moreover, a functional magnetic resonance imaging (fMRI) study has examined how adults with pedophilic interest and controls with sexual interest in adults respond to face stimuli of adults vs. children [18]. In the control group, adult compared to child faces caused higher brain responses in areas that have been linked to the processing of faces, i.e. the occipital and fusiform gyrus, and emotion processing, i.e. the ventrolateral prefrontal cortex. Interestingly, the pattern of effects was reversed in the group of men with pedophilia: the presentation of child compared to adult faces led to higher responses in face-related and emotion-related brain regions. Taken together, studies in men with pedophilia have shown aberrant neural processing of age and sexually relevant stimuli. This suggests a neurobiological mechanism underlying pedophilia, which provides opportunities to obtain objective biomarkers of sexual preference for prepubescent children. The aforementioned results relate exclusively to adults but did not involve juveniles to investigate the neural mechanisms underlying sexual age preferences early on.

In this study, we adopted the previously established face-viewing paradigm by Ponseti and colleagues (2014) to examine whether deviant neural processing of child and adult faces is found in juveniles with a sexual preference for childlike body schemes (PP group) [18]. We recorded high-density EEG and obtained attractiveness ratings of the presented child and adult face stimuli. Our main goal was to uncover differences in neural response patterns, as expressed in ERPs, to child and adult face stimuli between the PP group and a control group, comprising juveniles with age-adequate sexual preference (AA group). We expected group differences in ERPs to child face stimuli, especially at later processing stages reflecting higher level stimulus processing, and attractiveness ratings.

## Materials and methods

The study was conducted in accordance with the 2008 Declaration of Helsinki and ethical approval was obtained by the Ethics Subcommittee 2 of the Campus Virchow-Klinikum of the Charité-Universitätsmedizin Berlin (application number: EA2/064/15).

### Participants

All juveniles were enrolled in the context of a larger project (JUNIOR—Juveniles’ influences on sexual offense risk against children) that is coordinated by the Institute of Sexology and Sexual Medicine, Charité - Universitätsmedizin Berlin. The original plan was to recruit a juvenile control group from the general population. However, the ethical committee had concerns regarding this proceeding (due to the involvement of the potentially offensive sexual case history). Nevertheless, it provided approval for the recruitment of a juvenile control group with age-adequate sexual preference (AA group) from a specialized ward “Addiction of video gaming and pathological media use” of the Vivantes Klinikum im Friedrichshain (Berlin, Germany) where a routine sexual case history is obtained.

Twenty-six juveniles with a sexual preference for childlike body schemes (PP group) were recruited from the Berlin Project for Primary Prevention of Child Sexual Abuse by Juveniles (PPJ), which offers diagnostic and therapeutic help to juveniles between 12 and 18 years with sexual preference for prepubescent children in Germany since 2014 [9]. Sexual age preference was assessed during a comprehensive clinical assessment by experienced and specially trained psychologists. Diagnostics included questionnaires and interviews with focus on masturbatory fantasies and fantasies causing sexual climaxes as well as sexual behavior and were supported by Viewing Time measurements [9]. Furthermore, an IQ-test (Wechsler Intelligence Scale for Children WISC-IV [23] or Wechsler Adult Intelligence Scale WASC-IV [24]) and a screening for psychiatric comorbidities (Diagnostic System for Mental Disorders in Childhood and Adolescence (DISYPS-III) [25]) were conducted. Only self-motivated juveniles with sexual interest for prepubescent children, who have not committed any CSO, or self-motivated juveniles with this sexual age preference, whose CSOs are undetected and/or unprosecuted (so-called “Dunkelfeld”) are offered a treatment program to enhance behavioral control within the PPJ [26].

Twenty-two juveniles with age-adequate sexual preference (AA group) were recruited as a control group from the specialized ward “Addiction of video gaming and pathological media use” of Vivantes Klinikum im Friedrichshain, located in Berlin, Germany. Besides addiction-specific interviews, and an IQ-test (WISC-IV[23], WASC-IV[24], or Culture Fair Test-Revised CFT 20-R [27]), diagnostics also included a comprehensive interview on sexual case history encompassing sexual fantasies and sexual behavior. The final control group comprised individuals with pathological use of internet and/or video games (according to other impulse disorder, ICD-10 diagnosis F63.8) (*n* = 6), with excessive consumption of internet and video games, but not fulfilling all ICD-10 criteria for a diagnosis (*n* = 4), and with moderate consumption of internet and video games (*n* = 12).

All participants were male, Caucasian and were compensated for participation (85–100€). The following inclusion criteria were applied to both groups: age between 14 and 18 years (18 years included); male; IQ of 100 ± 30; no alcohol or substance abuse; no autism spectrum disorders, whereas patients with other comorbidities could be included. Groups were matched for age, IQ and most of ICD-10 coded comorbidities, and were not matched for sexual gender preference, exclusiveness and handedness assessed by Edinburgh Handedness Inventory [28] (Table 1). One juvenile of the PP group was treated with Naltrexone, Lisdexamfetamine and Atomoxetine, and one juvenile of the AA group was treated with Methylphenidate. Both were diagnosed with hyperkinetic disorder. Another juvenile (AA group) with symptoms of depression was treated with Escitalopram (Table 1).

**Table 1:** Demographic and clinical information about study participants

	PP group	Control group	*t* test
Age [years], *M* (SD)	16.24 (1.48)	16.32 (1.21)	*t*(45) = − 0.197, *p* = 0.845, *d* = 0.059
IQ^a^, *M* *(SD)*	97.75 (15.44)	103.57 (9.80)	*t*(39.429) = − 1.529, *p* = 0.134, *d* = 0.487
Hetero-/homo-/bisexual	12/5/8	22/0/0	
Exclusive/non-exclusive	12/13	22/0	
Right-/left-/both-handed	25/0/0	18/3/1	

	PP group	Control group	Mann–Whitney test
Comorbidities			
Other habit and impulse disorders	1^b^	6^c^	*U* = 211, *Z* = − 2.212, *p* = 0.027
Hyperkinetic syndrome	8	2	*U* = 212, *Z* = − 1.894, *p* = 0.058
Mild and moderate depression episode	0	4	*U* = 225, *Z* = − 2.205, *p* = 0.027
Somatization disorder	0	1	*U* = 262.5, *Z* = − 1.066, *p* = 0.286
Psychological and behavioral disorders associated with sexual development and orientation	1	0	*U* = 264, *Z* = − 0.938, *p* = 0.348
Conduct disorders	1	0	*U* = 264, *Z* = − 0.938, *p* = 0.348
Mixed disorders of conduct and emotions	1	0	*U* = 264, *Z* = − 0.938, *p* = 0.348
Emotional disorders with onset specific to childhood	1	0	*U* = 264, *Z* = − 0.938, *p* = 0.348
Disorders of social functioning with onset specific to childhood and adolescence	1	0	*U* = 264, *Z* = − 0.938, *p* = 0.348
Obesity due to excess calories	1	0	*U* = 264, *Z* = − 0.938, *p* = 0.348

PP group = juveniles with a sexual preference for prepubescent children. Control group (AA group) = juveniles with age-adequate sexual preference

^a^Data missing for each one participant of PP and AA group

^b^ICD-10 F63.8 code in terms of an impulsive behavior

^c^ICD-10 F63.8 code in terms of pathological use of internet and/or video games

All participants (and if underage, also the person who has custody of the participant) gave written informed consent, had normal or corrected to normal vision and no record of neurological disorders. One participant of the PP group was excluded due to a relatively high number of undetected catch trials (> 10%) in the experiment.

### Stimuli

Photographs depicting child faces were taken from the database Child Affective Facial Expression set (CAFE), which includes an ethnically diverse group of 2- 8 year-old children with six emotional facial expressions and a neutral facial expression [29]. Pictures of adult faces were provided by the Chicago face database, which consists of standardized photographs of Black and White males and females between the ages of 18–40 years [30] and the Karolinska Directed Emotional Faces database, which comprises 70 White amateur actors from 20 to 30 years old [31]. All photographs were standardized with the free image processing tool GIMP 2.8 (www.gimp.org). Each face was manually cut along the head (including ears and hair in line with the chin), converted to greyscale, brought to the same size and the same central position on a grey background (RGB: 204, 204, 203, visual angle = 6.9° × 6.1°). The photographs consisted of 30 women, 30 men, 30 girls, and 30 boys, each expressing three different emotions (neutral face, happy face, and angry face). This resulted in 360 different photographs. In addition, 70 pictures with neutral, non-human content and which have been shown to induce only low levels of arousal (1.72–3.99) and valence (4.25–5.93) were selected from the International Affective Pictures Systems Database (IAPS) as catch trials [32]. Pictures were equally sized and converted to greyscale. All photographs and neutral pictures were equalized in luminance with the SHINE toolbox [33].

### Experimental design

**Fig. 1:**
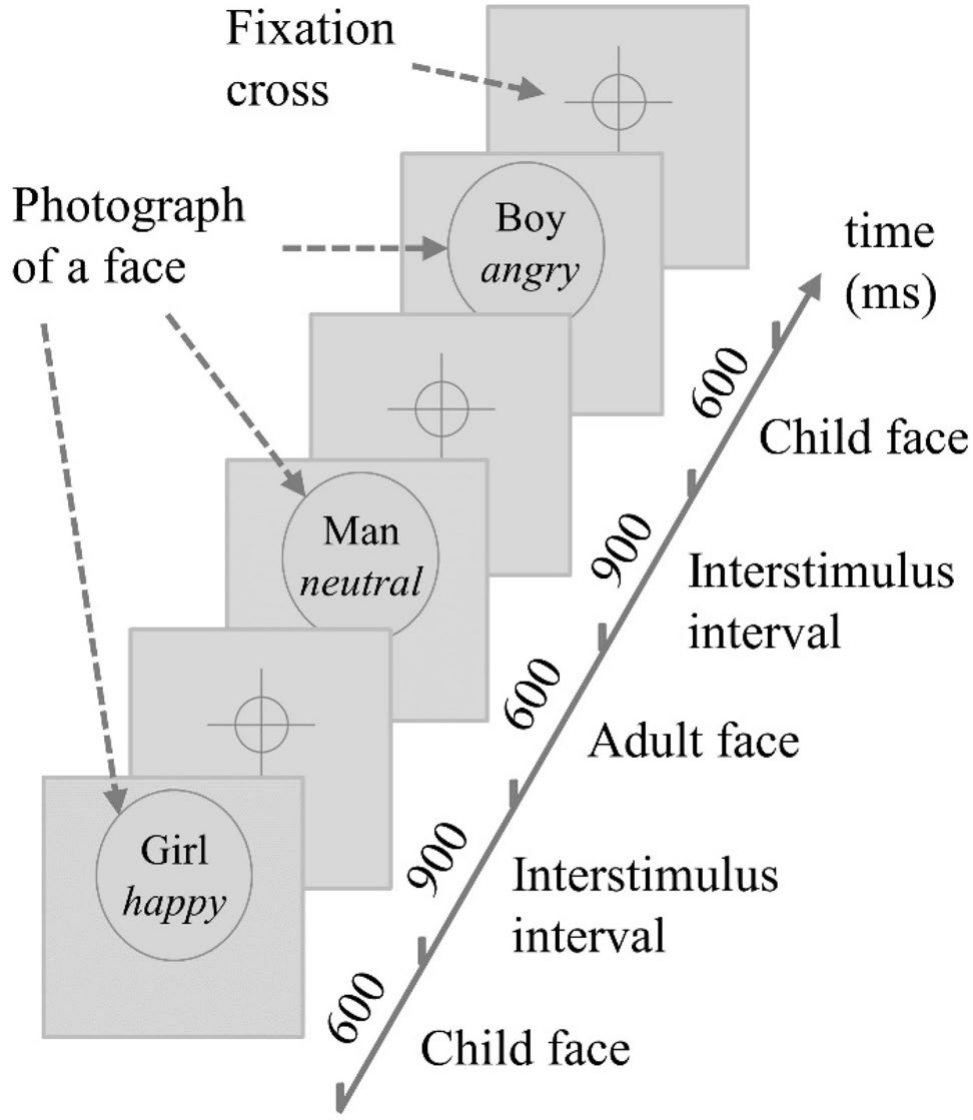
Illustration of the passive face-viewing paradigm. Photographs of children and adult faces with neutral, happy, and angry expressions were presented in random order. To ensure visual attention, occasional photographs from the IAPS database were presented (not illustrated) and participants were instructed to respond to these catch trial stimuli by a button press. The interstimulus interval varied between 700 and 1100 ms (average 900 ms). For reasons of copyright protection, placeholders are shown instead of the original photographs

The experiment consisted of 1296 (100%) trials, including 216 (20%) catch trials and 1080 (80%) critical trials with photographs of faces. Each photograph of a face was presented three times. The trials were pseudo-randomized, to avoid more than three repetitions of the same age, emotion and gender. Each facial picture was presented for 600 ms, and the interstimulus interval (ISI) varied between 700 and 1100 ms (average 900 ms), during which a fixation cross was presented approximately at eye level to minimize eye movements (Fig. 1).

The 1296 trials were presented in eight blocks of 162 trials each. Every block had a duration of about 4 min. Participants had the opportunity to take short breaks after every second block. They were asked to relax during the experiment and to move and blink as little as possible. No response was required when a face stimulus was presented. However, participants were instructed to press the space bar on a keyboard when a catch trial was presented. The presentation of catch trials ensured that participants attended to the visual stimuli. If a catch trial was missed, the stimulation continued after 2000 ms. All stimuli were presented in a dimly lit, sound-attenuated electrically shielded chamber (located at the St. Hedwig Hospital, Berlin, Germany) on a CRT monitor with a background luminance of 21 cd/m^2^. Stimuli and manual responses were controlled using Presentation® software (Version 18.0, Neurobehavioral Systems, Inc., Berkeley, CA, www.neurobs.com).

After the EEG experiment, participants were asked to complete two post-rating tasks. While all neutral faces of men, women, girls, and boys (in total 120) were shown, participants were first asked to assign gender (female, male) and age (child, adult) to the faces. Then, they were asked to classify the attractiveness of the face on a four-step scale (“very attractive—somewhat attractive—less attractive—not attractive”). The reason for obtaining ratings only for neutral faces was to avoid the confounding influence of possible general differences in facial emotion processing between groups.

### Analysis of behavioral data

Statistical analyses were conducted with IBM SPSS Statistics Version 25.0 (IMB Corp., Armonk, NY).

#### Personal data

Independent *t* tests were calculated to prove matched groups for age and IQ. Mann–Whitney tests were conducted to analyze the distribution of ICD-10 diagnoses between PP group and AA group (Table 1).

#### Catch trials

For each participant, the mean and standard deviation of missed catch trials were calculated and put in relation to the total amount of catch trials. An independent *t*-test was computed to compare missed catch trials.

#### Gender, age and attractiveness ratings of faces

The correct allocation of neutral face stimuli to sex and gender was summarized in relation to all participants. The mean and standard deviation of attraction classifications was calculated separately for PP and AA group. Then, a 2 × 2 repeated measure analysis of variance (ANOVA) was conducted with the between-factor group (PP group vs. AA group) and the within factor age category (child vs. adult faces). If a significant effect was found in the ANOVA, Bonferroni-corrected *t* tests were computed to follow up on the effects. As the PP group consisted of juveniles with exclusive and non-exclusive sexual preferences, we further conducted a 2 × 2 ANOVA with the between-factor group (exclusive vs. non-exclusive PP) and the within-factor age category (child vs. adult faces).

### Acquisition and processing of EEG data

EEG data were recorded with a high-density 128-channel active EEG system (EasyCap, Herrsching, Germany), including one horizontal and one vertical EOG electrode placed below and lateral to the right ocular orbit to register eye movements using Brainamp DC amplifiers (Brainproducts, Gilching, Germany). Recordings were made against nose reference at a sampling frequency of 1000 Hz and with a passband of 0.016–250 Hz.

EEG data preprocessing and data analysis were conducted in MATLAB (MathWorks, Natick, MA, USA) using EEGLAB (http://www.sccn.ucsd.edu/eeglab) [34], FieldTrip (http://www.ru.nl/fcdonders/fieldtrip) [35] and customized scripts. First, data were filtered offline using windowed sinc FIR filters [36] (high pass: 1 Hz, low pass: 125 Hz, notch: 49–51 Hz). Furthermore, data were down sampled to 500 Hz and epochs from − 0.5 to 0.6 ms relative to visual stimulus onset were extracted from the data. Epochs with large artefacts were removed by visual inspection (*M* removed trials = 135.33 out of 1080, SD = 65.75, PP group: *M* removed trials = 151.84, SD = 62.90, AA group: *M* removed trials = 114.70 trials, SD = 63.37) at an approximal equal rate from all subcategories (sex, age and emotional face expressions). To further correct for EOG artefacts (blinks, muscle activity) and strong cardiac activity, independent component analysis (ICA) was conducted (Runica) [37]. On average 2.06 ICA components (SD = 0.86, PP group: *M* removed components = 2.20, SD  = 0.85, AA group: *M* removed components = 1.91, SD  = 0.85) were removed. Channels with extremely high artifacts were interpolated with distance interpolation (PP group: *M* removed electrodes = 5.12 electrodes, SD  = 1.80, AA group: *M* removed electrodes = 4.68, SD  = 1.66). The EOG channels were not included in the further analysis of ERPs. For the analysis of ERPs, epochs were low-pass filtered (45 Hz, using a two-pass fourth-order Butterworth filter), detrended, and baseline corrected using an interval from − 0.5 to 0.1 s prior to stimulus onset.

### Analysis of evoked brain activity

In accordance with previous studies, one anterior [38–41] and one posterior [40, 42–44] region of interest (ROI) were included in the data. The posterior ROI comprised 30 bilateral symmetric electrodes, encompassing cortical regions over the occipital cortex. This ROI was selected for the analysis of early ERP components (< 200 ms). The frontal ROI encompassed seven bilateral symmetric electrodes around the Fz-electrode. This ROI was selected for the analysis of late ERP components (> 320 ms). To define the exact relevant time of interest (TOI), data of all participants and all conditions were averaged separately for the frontal and the occipital ROI. The maximum between 0 and 200 ms was calculated for the occipital ROI to determine TOI for early ERPs component. Based on the average across participants and conditions, this TOI was defined as ± 10 ms around the maximum of 112 ms, namely 102–122 ms, corresponding to the visual evoked P1 component [44]. The TOI for the later component was defined by calculating the minimum between 320 and 600 ms for the frontal ROI. This time window was selected based on the average across participants and conditions. Not only one minimal peak (i.e. maximum negative amplitude) was found, but two (340 ms and 426 ms), so that the later TOI was defined between the two negative peaks, i.e. 340–426 ms, during which a negative deflection was found. In the following, we will refer to this deflection as late frontal negativity (LFN).

In the next step of the analysis, EEG data were exported separately for each participant and condition for the frontal ROI and TOI and for the occipital ROI and TOI. The assumption of a normal data distribution (across participants) was confirmed by Shapiro–Wilk test for both components and for child and adult faces (*p* > 0.05). Two 3 × 2 × 2 repeated measure ANOVAs with the between-subjects factor group (PP vs. AA group) and the within-subjects factors age (child vs. adult faces) and emotional expression (happy vs. angry vs. neutral) were calculated. The first ANOVA was calculated for the early time window, i.e. P1 component, at the occipital ROI. The second ANOVA was calculated for the late time window, i.e. LFN, at the frontal ROI. To correct for multiple comparisons across the two ROIs, the alpha level was adapted to  0.05/2 = 0.025. If an effect in the ANOVA was significant, paired follow-up *t*-tests with Bonferroni correction for multiple comparisons were computed. In case of a non-significant three-way interaction, we reduced the ANOVA to a 2 × 2 design with the between-subjects factor group (PP vs. AA group) and the within-subjects factors age (child vs. adult faces). Cohen’s d [45, 46] and partial eta-squared [47] were calculated as measures of effect size.

### Source projection of evoked brain activity

To investigate the cortical sources of the observed ERP responses in the early and late time windows, we followed the approach previously published in our research group [49]. We performed source localization using a linearly constrained minimum variance (LCMV) beamformer algorithm [48]. A leadfield was generated using a realistic three-shell boundary-element volume conduction model based on the MNI standard brain (MNI; http://www.mni.mcgill.ca) for each grid point in the brain on a regular 10-mm grid. We first constructed a common spatial filter across all participants and all conditions from the covariance matrix of the averaged single trials at electrode level and the respective leadfield. The use of a common spatial filter for all data guaranteed that differences in source space activity could be ascribed to power differences in the different conditions and groups and not to differences between filters. The lambda regularization parameter was set to 5%, to compensate for potential rank reduction during preprocessing. A baseline correction was performed for each time window and condition. To this end, the activity in the respective baseline interval (P1: − 122 to − 102 ms; LFN: − 426 to − 340 ms) was first subtracted from the post-stimulus interval of interest, and the resulting difference was then divided by the average baseline activity [49].

## Results

### Behavioral data

#### Catch trials of the main experiment

On average, individuals of the PP group missed 1.40 (SD = 2.42) catch trials and individuals of the AA group missed 0.73 (SD = 2.33, PP group vs. AA group: *t*(45) = 0.968, *p* = 0.338, *d* = 0.289). One PP was excluded from the further analysis because he missed more than 10% of catch trials.

#### Gender and attractiveness ratings of faces

**Fig. 2:**
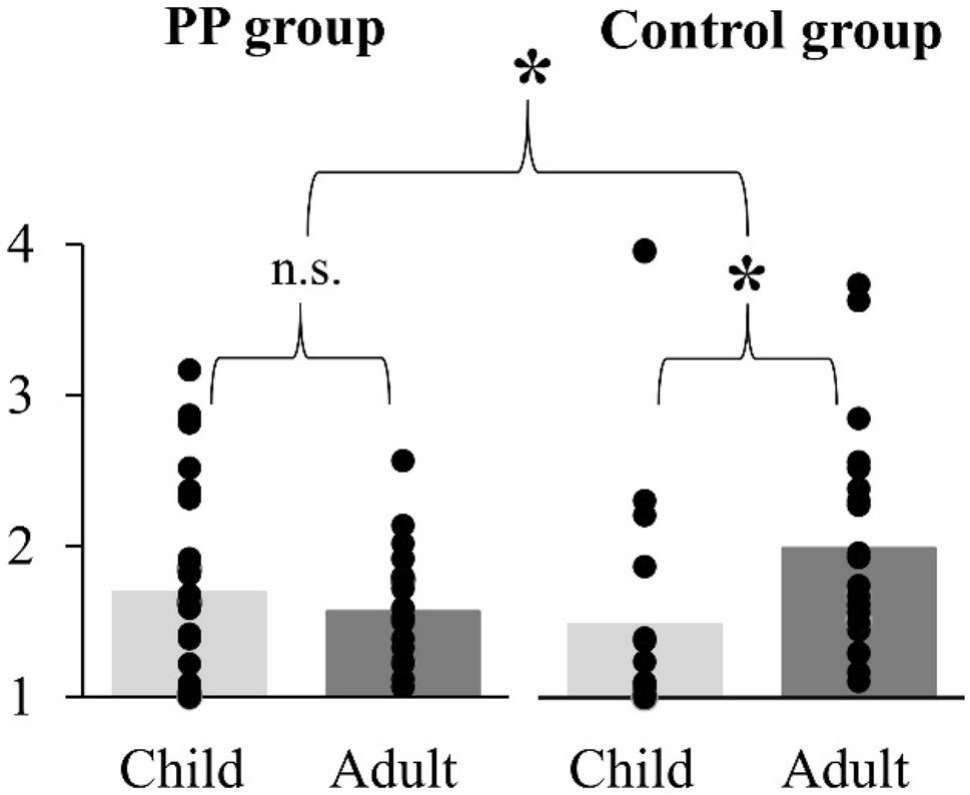
Attractiveness ratings for faces (from very attractive = 4 to not attractive = 1). Juveniles with a sexual preference for prepubescent children (PP group, left panel) and the control group (right panel). Significant differences in attractiveness ratings between children and adult faces were specifically found in the control group

The vast majority, i.e. 94%, of the neutral face stimuli had a gender and age detection accuracy of more than 70%. Importantly, there were no differences in gender and age rating accuracy between groups (*t* tests (Bonferroni-corrected alpha = 0.05/4): PP group vs. AA group for girls: *t*(45) = 1.613, *p* = 0.114, *d* = 0.481, for boys: *t*(45) = − 0.093, *p* = 0.926, *d* = 0.028, for women: *t*(45) = 1.945, *p* = 0.058, *d* = 0.580, for men: *t*(45) = 0.838, *p* = 0.406, *d* = 0.250). On a scale from 1 (unattractive) to 4 (very attractive), faces were ranked with a mean value of 1.69 (SD  = 0.70) (Table 2 and Fig. 2). The ANOVA revealed a significant main effect of age [*F*(1, 45) = 4.693, *p* = 0.036, (*η*_p_^2^ = 0.094)] and a significant interaction between age and group [*F*(1, 45) = 13.440, *p* = 0.001 (*η*_p_^2^ = 0.230)]. The significant interaction demonstrates group differences in the ratings of child vs. adult faces. Follow-up *t* tests (Bonferroni-corrected alpha = 0.05/2) showed no significant difference between ratings for child vs. adult faces in the PP group [*t*(24) = 1.061, *p* = 0.299, *d* = 0.212], but significantly higher attraction ratings for adult vs. child faces in the AA group [*t*(21) =  − 4.159, *p* < 0.001, *d* = 0.887] (Fig. 2).

**Table 2:** Attraction ratings of faces within a four-step scale: very attractive (4)—somewhat attractive (3)—less attractive (2)—not attractive (1)

Ratings, *M* (SD)	PP group (*n* = 25)	Control group (*n* = 22)	Exclusive PP (*n* = 12)	Non-exclusive PP (*n* = 13)
Child faces	1.70 (0.66)	1.49 (0.90)	1.86 (0.70)	1.55 (0.61)
Adult faces	1.57 (0.38)	2.00 (0.74)	1.50 (0.45)	1.63 (0.31)

Depicted are mean and standard deviation. PP group = juveniles with a sexual preference for prepubescent children. Control group with age-adequate sexual preference

Analyzing individuals of the PP group with an exclusive and non-exclusive sexual preference separately, individuals with an exclusive sexual preference for prepubescents rated child faces as more attractive than adult faces, while non-exclusive individuals rated in the opposite direction (Table 2). However, there was no significant condition effect for age [*F*(1, 23) = 1.405, *p* = 0.248 (*η*_p_^2^ = 0.058)] and no significant interaction between age and group [*F*(1, 23) = 3.370, *p* = 0.079 (*η*_p_^2^ = 0.128)].

### Evoked brain activity

#### P1-component

Comparing the amplitude of the P1 component between groups and conditions, there were main effects of age [*F*(1, 45) = 27.478, *p* < 0.001 (*η*_p_^2^ = 0.379)] and emotion [*F*(2, 90) = 3.943, *p* = 0.023 (*η*_p_^2^ = 0.081)]. Adult faces had stronger responses compared to child faces in both groups. Angry faces elicited overall larger P1 amplitudes compared to happy and neural faces, with exception for adult faces in AA group (Table 3).

**Table 3:** Mean P1 amplitudes (µv) in response to facial expressions with angry, happy, and neutral expressions

P1 amplitudes, *M* (SD)	PP group (*n* = 25)	Control group (*n* = 22)
Angry faces		
Child faces	4.18 (2.18)	4.41 (1.54)
Adult faces	4.63 (2.28)	4.86 (1.73)
Happy faces		
Child faces	4.06 (2.20)	4.25 (1.43)
Adult faces	4.50 (2.25)	5.04 (1.72)
Neutral faces		
Child faces	3.92 (2.14)	4.18 (1.59)
Adult faces	4.25 (2.22)	4.95 (1.81)

Mean LFN amplitudes are not listed because there was no significant effect of facial expression on this component. PP group = Juveniles with a sexual preference for prepubescent children. Control group with age-adequate sexual preference

**Fig. 3:**
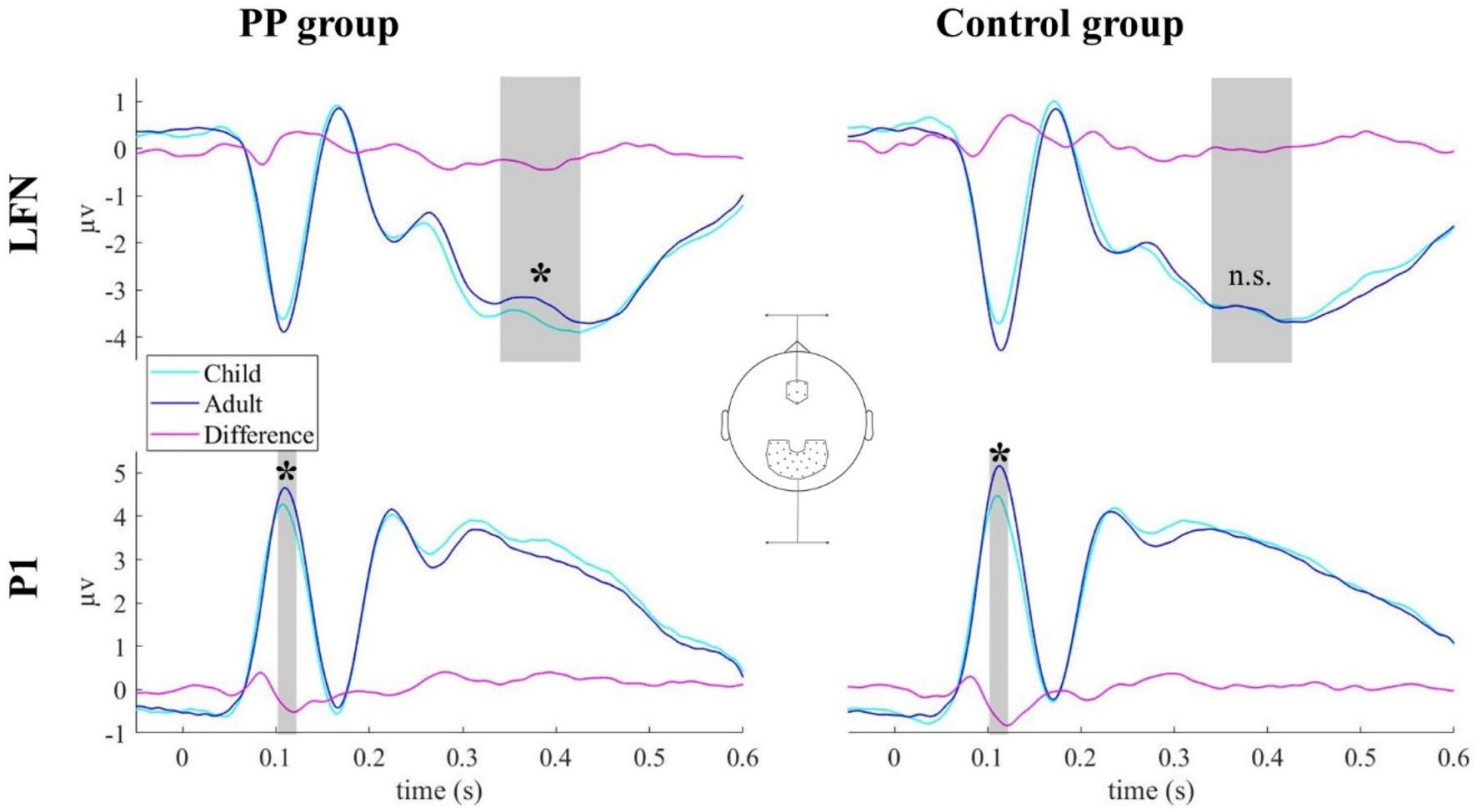
Event-related potential traces for the occipital P1 (bottom) and the frontal LFN (top) components for juveniles with a sexual preference for prepubescent children (PP group, left panel) and the control group (right panel). Group interactions were found for the LFN component. In the PP group frontal LFN amplitudes were significantly larger for child vs. adult faces, whereas no such difference was found in the control group (upper panel)

**Fig. 4:**
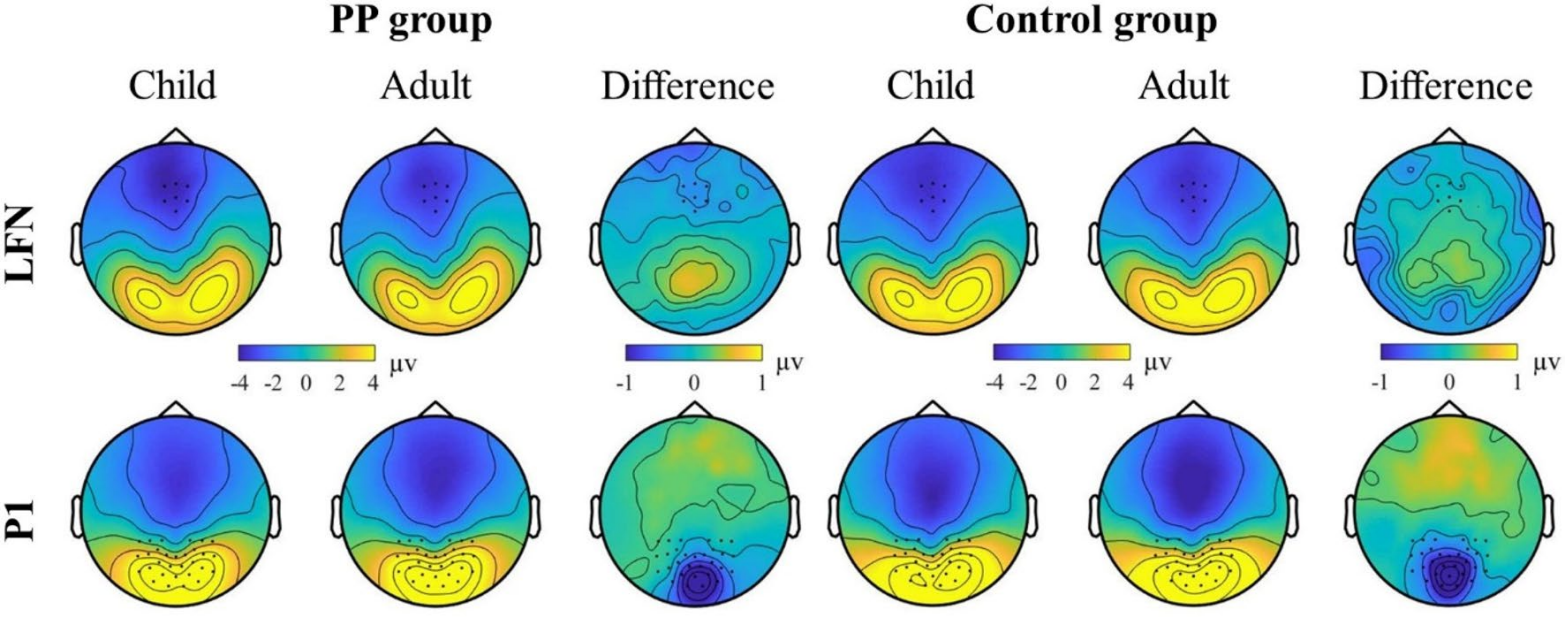
Topographic distributions of the P1 (bottom) and LFN (top) components in juveniles with a sexual preference for prepubescent children (PP group, left panel) and the control group (right panel)

There was no significant interaction between age, emotion and group [*F*(2, 90) = 1.247, *p* = 0.292 (*η*_p_^2^ = 0.027)], nor was there a significant interaction between emotion and group [*F*(2, 90) = 1.64, *p* = 0.200 (*η*_p_^2^ = 0.035)], and finally no significant interaction between age and group [*F*(1, 45) = 1.619, *p* = 0.210 (*η*_p_^2^ = 0.035)]. As planned, a follow-up 2 × 2 ANOVA was conducted, in which the factor emotion was omitted. For this ANOVA there was also no significant interaction between age and group [*F*(1, 45) = 1.613, *p* = 0.211 (*η*_p_^2^ = 0.035)]. ERP traces (Fig. 3) and topographic plots (Fig. 4), illustrate higher P1 amplitudes to adult compared to child faces in both groups.

**Fig. 5:**
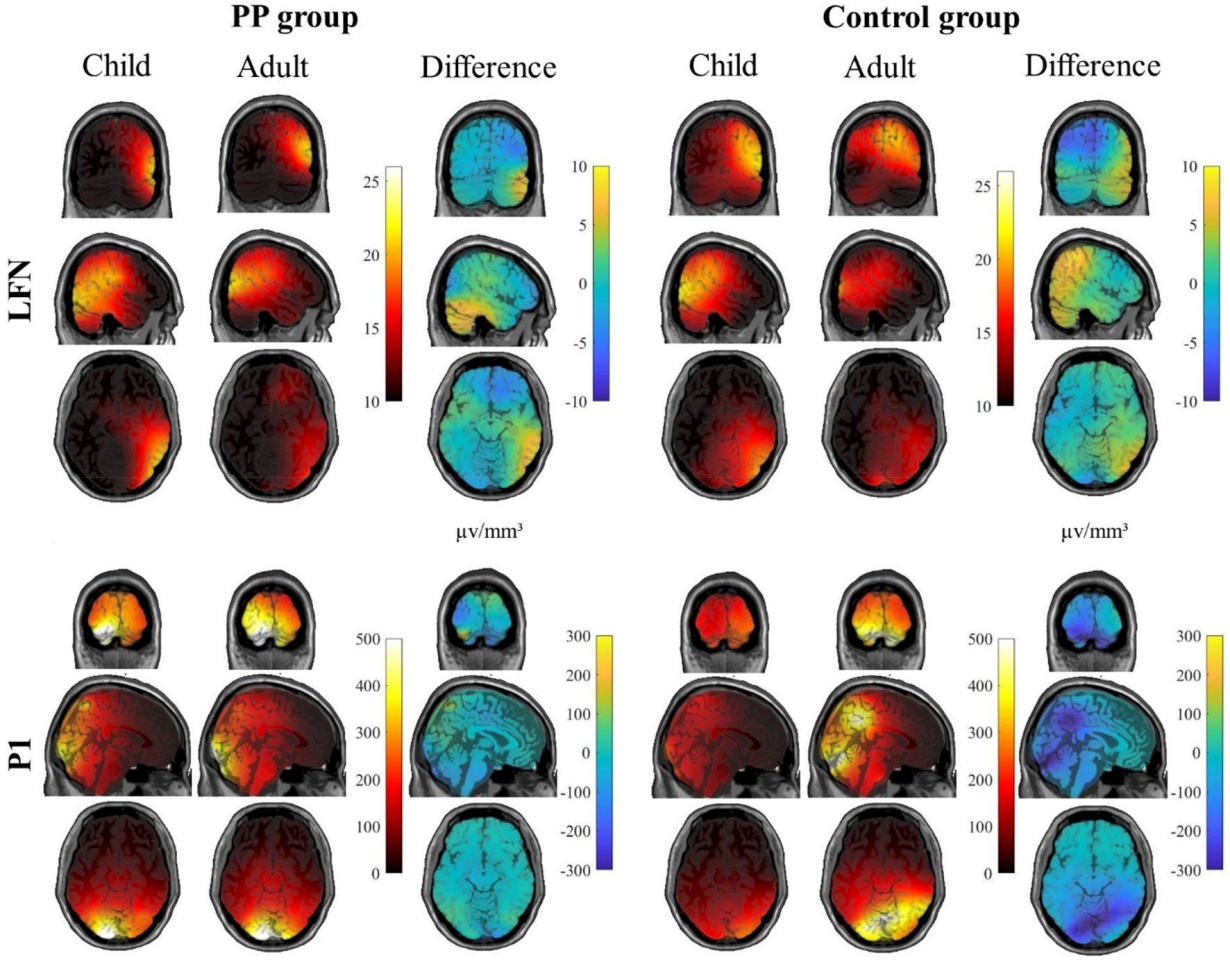
Source projection of the P1 (bottom) and LFN (top) components for juveniles with a sexual preference for prepubescent children (PP group, left panel) and the control group (right panel)

The source level analysis of the P1 component revealed sources primarily in the occipital lobe (Fig. 5). Notably, a negative difference between child and adult faces was visible for both groups in the occipital cortex.

#### Late frontal negativity (LFN)

As data were tested in two time windows,  the alpha level was adapted to 0.05/2. Comparing the amplitude of the LFN component between groups and conditions, there was no main effect for emotion [*F*(2, 90) = 0.603, *p* = 0.550 (*η*_p_^2^ = 0.013)] or age [*F*(1, 45) = 4.944, *p* = 0.031 (*η*_p_^2^ = 0.099)]. In addition, there was no significant interaction between emotion and group [*F*(2, 90) = 0.313, *p* = 0.732 (*η*_p_^2^ = 0.007)] and age, emotion, and group [*F*(2, 90) = 2.019, *p* = 0.139 (*η*_p_^2^ = 0.043)], nor a significant interaction between age and group [*F*(1, 45) = 5.192, *p* = 0.027 (*η*_p_^2^ = 0.103)].

As planned, a follow-up 2 × 2 ANOVA was conducted, in which the factor emotion was omitted.

Notably, this ANOVA revealed a significant interaction between group and age [*F*(1, 45) = 5.349, *p* = 0.025 (*η*_p_^2^ = 0.106)]. Follow-up *t*-tests (Bonferroni corrected alpha = 0.05/2) showed a significantly stronger frontal negativity for the PP group while watching child faces compared to adult faces [*t*(24) =  − 3.170, *p* = 0.004, *d* = 0.634]. In contrast, individuals of the AA group did not show differences in LFN amplitudes following child vs. adult faces [*t*(21) = 0.045, *p* = 0.965, *d* = 0.010].

At source level, activation of primary and higher-order visual areas was found during the time window of the LFN (Fig. 5). A positive difference between child and adult faces was observed in parietal areas (for both PP and AA group) and a negative difference was found in frontal areas (especially for the PP group).

**Fig. 6:**
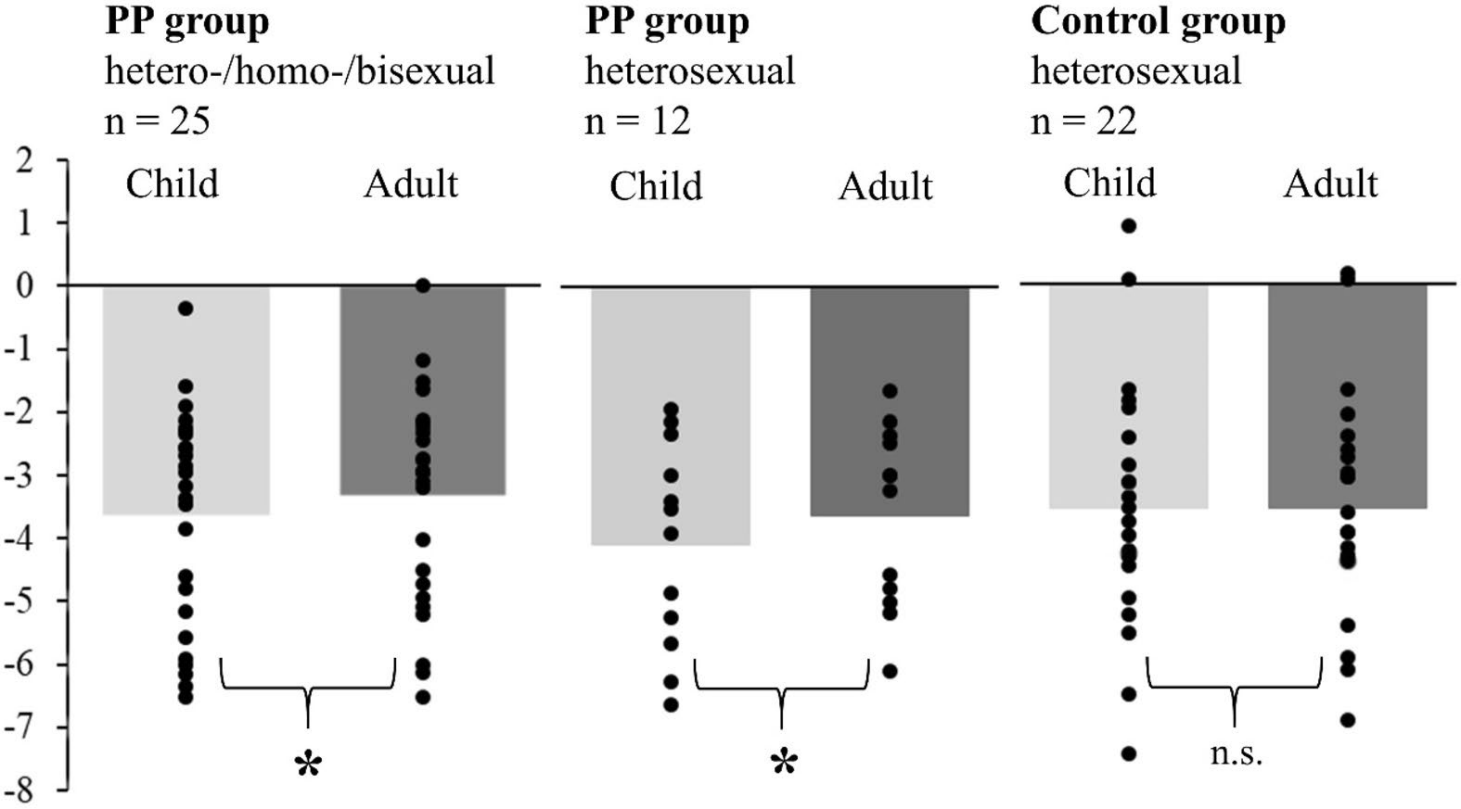
Mean of frontal LFN amplitudes for complete juveniles with a sexual preference for prepubescent children (PP group, *n* = 25, left panel), a subgroup of heterosexual juveniles with a sexual preference for prepubescent children (heterosexual PP group, *n* = 12, middle panel) and heterosexual individuals from the control group (*n* = 22, right panel). No LFN amplitude differences between child vs. adult faces were found in the control group, whereas significant higher LFN amplitudes for child faces were found in the PP group (both for all participants and for only heterosexual participants of this group)

Being aware of the heterogenous sex preference in the PP group (12 heterosexual, five homosexual, eight bisexual participants), it was important to explore that this heterogeneity did not substantially contribute to the observed effect on the LFN. Therefore, a separate statistical analysis was conducted for only heterosexual participants, namely the subgroup of 12 heterosexual PP and 22 heterosexual AA group. In this analysis, the mean amplitude of LFN for child vs. adult faces was larger for the subgroup of only heterosexual PP juveniles (*n* = 12), similar to the main analyses of the amplitude of LFN in the complete PP group (*n* = 25). However, there was no difference between child vs. adult faces for the AA group (Fig. 6).

Further *t* tests confirmed this finding: There was a significant difference between child vs. adult faces for the complete PP group [*t*(24) =  − 3.170, *p* = 0.004, *d* = 0.634], for the subgroup of 12 heterosexual PP [*t*(11) =  − 2.694, *p* = 0.021, *d* = 0.778], but there was no difference for the AA group [*t*(21) = 0.045, *p* = 0.965, *d* = 0.010]. Thus, the heterogeneity of sex preferences within the PP group did not represent a confounding factor. In a more exploratory analysis, we examined whether there are relationships between ERP amplitudes (i.e. P1 and LFN) and attractiveness ratings of neutral faces. To this end, we computed Spearman’s Rho correlations between ERP amplitudes and attractiveness ratings separately for each study group. This analysis revealed no significant relationships (all *p* values > 0.374).

## Discussion

In this study, we examined the processing of child and adult faces in a group of juveniles with a sexual preference for prepubescent children (PP group) and a control group, comprising juveniles with age-adequate sexual preference. In the control group, attractiveness ratings were higher for adult faces vs. child faces. No such difference was found in the PP group. Investigating the neural responses to child vs. adult face stimuli, as reflected in event-related potentials (ERPs), we found group differences at later (340–426 ms) processing stages. Later ERPs in the PP group were larger for child faces vs. adult faces, while no such difference was found in the control group.

Behavioral data of attractiveness ratings revealed group differences in the ratings of child vs. adult faces. Attractiveness ratings in the control group corresponded to the information given in the sexual case history: members of the control group preferred adults as compared to children. No significant differences in attractiveness ratings between child vs. adult faces were found in the PP group. Notably, the EEG recordings and face attractiveness ratings took place at a different location and with different experimenters, compared to their accustomed environment of therapy within the framework of Berlin Project for Primary Prevention of Child Sexual Abuse by Juveniles (PPJ). Therefore, it is possible that members of the PP group felt insecure about openly admitting their attraction to child faces and provided socially desirable answers. Furthermore, the PP group was composed of almost equal parts of exclusive, i.e. juveniles with sexual preference only for minors, and non-exclusive juveniles, i.e. individuals with sexual interest in minors and majors. Although we did not find group differences between exclusive PP and non-exclusive PP, perhaps due to a lack of statistical power, exclusive PP numerically rated child faces as more attractive compared to adult faces, whereas non-exclusive PP rated the opposite way, mitigating the ratings of exclusive PP. Thus, it could be that a larger sample of exclusive PP individuals would have shown significant higher attraction ratings for child vs. adult faces. In summary, our analysis of attractiveness ratings revealed differences between study groups, indicating altered perception of child vs. adult faces in the PP group.

The analysis of evoked brain responses to child vs. adult faces revealed effects for earlier (102–122 ms) and later (340–426 ms) ERP components. For early ERP components, presumably reflecting feature integration and face categorization [50], we observed larger P1 amplitudes for adult compared to child faces in both groups. Source localization showed that this effect involved occipital areas. Thus, in line with findings from previous ERP studies, differences in the visual processing of face stimuli depicting older and younger individuals occurred within 200 ms after stimulus presentation [43, 51]. Recently, it has been found that women show enhanced neural responses for child faces vs. adult faces, whereas the opposite was found in men, who showed enhanced brain responses to adult faces [43]. This latter finding fits with the enhanced early ERPs to adult faces in our study, which included only male participants. Moreover, resembling other previous observations [43, 52–54], facial expressions could be differentiated in both groups: emotional expressions (here: anger even more than happiness) elicited stronger amplitudes compared to neutral faces. This supports the notion that emotional face discrimination occurs during earlier stages of face processing, as those emotional expressions require rapid social and emotional responses [54]. Previously, Wiese and colleagues (2014) proposed that the attractiveness of faces does not modulate early face processing, which could explain why we did not find group differences at earlier processing stages [55]. Taken together, our results are concordant with previous findings in adult male participants, revealing early-enhanced brain responses to adult vs. child faces in juveniles.

In contrast to earlier ERPs, we did find group differences for late ERPs to child faces vs. adult faces. Whereas juveniles of the control group showed no late ERP differences between child vs. adult face stimuli, individuals of the PP group showed larger LFN amplitudes for child faces vs. adult faces stimuli. In line with some previous studies in juveniles and young adults [56–59] [but see: [53], we did not find differences in late ERPs between the different emotional facial expressions. Notably, most previous studies that reported emotion-related modulations of face processing have investigated adults [42, 53, 60]. Hence, we argue that the current setup, i.e. passive viewing of faces in juveniles, did not lead to substantial amplitude differences between neutral, happy, and angry faces. More importantly, our findings of emotion-independent amplitude differences in child faces vs. adult faces in the PP group are in line with fMRI reports showing that face processing is modulated by sexual gender orientation [61] and sexually preferred age [18]. Moreover, they align with previous EEG studies demonstrating that the processing of attractive faces is reflected in enhanced late ERPs [39, 55, 62, 63]. For instance, van Hooff and colleagues (2010) showed that attractive faces do not simply modulate neural responses because of general sexual attraction, but can also be differentiated in terms of gender-specific sexual preference [39]. Heterosexual men showed increased responses for attractive women’s faces in later ERP components [39], similar to our findings of sexual age preferred modulation of longer latency ERPs in the PP group. In another study, Knott and colleagues (2016) examined the processing of sexual stimuli in a group of men with pedophilic interest and found attenuated frontal ERPs to adult erotic pictures at 200–450 ms [22]. In our study, we observed aberrant ERPs to child faces at frontal electrodes in juveniles with a sexual preference for childlike body schemes at a similar latency. Thus, late ERPs seem to be modulated by sexual gender or age preference in adults and juveniles. Source projection of our finding suggested an involvement of higher order visual areas, parietal and frontal brain regions. Ponseti and colleagues (2014) showed an involvement of similar brain structures, i.e. the inferior occipital gyrus, fusiform gyrus, left ventrolateral prefrontal cortex, by men with pedophilic interest when viewing sexually immature faces, and by teleiophilic control participants when viewing sexual mature faces [18]. Another fMRI study in adults reported activation of visual cortex, limbic systems and prefrontal cortex in response to male and female faces [61]. Interestingly, sexually preferred gender enhanced brain activation in orbitofrontal cortex and mediodorsal nucleus of thalamus is shown for female faces by heterosexual men and homosexual women and for male faces by heterosexual women and homosexual men. Hence, differential brain activation patterns have been associated with sexual age and sexual gender preference. In summary, our study revealed aberrant long latency processing of child faces vs. adult faces in the PP group, which could reflect an enhanced attentional capture towards the child faces in the PP group.

Some limitations of the study should be discussed. First, the patient group was relatively heterogeneous. As we included not only exclusive PP (*n* = 12), but also non-exclusive PP (*n* = 13), the effect of enhanced long-latency ERPs to child faces may be diminished, as non-exclusive PP are interested in both children and adults. Thus, we would have expected even larger effects on late ERPs in a more homogenous sample of exclusive PP individuals. Notably, this was partially the case when the 12 exclusive PP individuals were analyzed separately (Table 2). Second, the study groups were not matched for sexual gender orientation. The control group comprised only heterosexual individuals, whereas juveniles with sexual preference for either male (*n* = 5), female (*n* = 12) or both sexes (*n* = 8) were included in the PP group. Due to the relatively small subgroups of sexual gender orientation in PP, we only considered the sexual age preferences and disregarded the sexual preferred gender orientation in our main analyses. However, significant late ERP differences between child and adult face processing were also found when only the subgroup of heterosexual PP was investigated (Fig. 6). Nevertheless, future studies should recruit a larger sample of PP participants and control participants with matched sexual gender preference. This would allow a more systematic examination of the influence of the sexual preferred age and gender, which is important, as male offenders with pedophilic interest and male victims have been identified as a risk factor for recidivistic offenses [64–68]. Third, the control group comprised individuals with moderate to pathological consumption of video gaming or media use. The advantage of studying this group was the extensive sexual case histories. However, there could also be a confounding impact of pathological video gaming and media use. Deficits in early face processing or unconscious facial emotional processing have been previously reported in patients with internet gaming disorders [69] and in excessive internet users [70, 71]. As the control group contained more juveniles without the diagnosis of other impulse disorder (*n* = 16) compared to only few juveniles with a pathological use of internet and/or video games (*n* = 6), we propose that the impact on the processing of child face and adult face stimuli should be small. Fourth, some of the participants had psychiatric comorbidities and were medicated. However, only two participants (one in each group) were taking psychoactive medication to treat hyperkinetic symptoms and one additional participant of the control group was taking antidepressant medication. The possible impact of attentional deficit (hyperactivity) syndrome (ADHD) on face processing has been discussed [72], but mostly without showing an impact on early emotional face processing [73–75]. Furthermore, medication with Methylphenidate seems to normalize neural activity and to improve emotional recognition [72]. Finally, major depression modulated the N170 component in only one of four studies [76]. Apart from depression episodes and other habit and impulse disorders (F.63.8), the two study groups were matched for comorbidities (see Table 1). In line with the aforementioned literature, we do not expect that our findings are explained by the comorbidities or the involvement of three juveniles (out of 47) who were taking medication. To summarize, while our study has some limitations, we consider it unlikely that they substantially account for the findings of our study.

## Conclusion

This study provides empirical evidence for aberrant neural processing of child face stimuli in a group of juveniles with a sexual preference for prepubescent children. Our findings could reflect an enhanced late attention capture of face stimuli depicting members from the sexually preferred age group in juveniles with a sexual preference for prepubescent children. The study expands our understanding of the neural foundations underlying sexual interest in pre- and early pubescent children. It also provides a promising novel pathway for the development of more objective diagnostic biomarkers of sexual responsiveness to childlike body schemes in juveniles.
